# Multidisciplinary Management and Clinical Outcomes in Amebic Liver Abscess: A Prospective Study From a Tertiary Care Center

**DOI:** 10.7759/cureus.84094

**Published:** 2025-05-14

**Authors:** Rehman Munilal Mohammad, Srinivas Nalabothula, Naren Surapaneni, Tribhuneswari Marreddy, Krishna Vasist Popuri

**Affiliations:** 1 General Surgery, Dr. Pinnamaneni Institute of Medical Sciences and Research Foundation, Vijayawada, IND; 2 Medicine and Surgery, Dr. Pinnamaneni Institute of Medical Sciences and Research Foundation, Vijayawada, IND

**Keywords:** alcohol consumption, amebic liver abscess, clinical care outcomes, developing nations, entamoeba histolytica infection, hepatobiliary involvement, percutaneous catheter drainage, ultrasound-guided intervention

## Abstract

Background

An amebic liver abscess (ALA) remains a major public health concern in endemic regions, with alcohol consumption as a key predisposing factor. Despite advances in diagnostics and therapeutics, delayed diagnosis and inadequate management continue to contribute to patient morbidity. This study evaluates the clinical features, hepatobiliary involvement, and outcomes associated with a structured treatment approach in ALA.

Methods

This prospective observational study included 50 patients diagnosed with ALA at a tertiary care center between 2022 and 2024. Demographic data, clinical presentation, laboratory values, and imaging findings were assessed. Patients were treated with either antibiotics alone, percutaneous needle aspiration, or pigtail catheter drainage. Outcomes, including hospital stay, recurrence, and mortality, were recorded over a three-month follow-up period.

Results

The mean age was 45.76 ± 10.2 years, with a male predominance (44 patients, 88%). Alcohol use was observed in 49 patients (98%), followed by malnutrition in 24 (48%) and anemia in 21 (42%). Jaundice was present in 25 patients (49%), suggesting significant hepatobiliary involvement. Right lobe abscesses occurred in 47 patients (94%). Antibiotic therapy alone was effective in 20 patients (40%), while 13 (26%) required needle aspiration and 17 (34%) underwent pigtail catheter drainage. Patients managed with pigtail drainage demonstrated more rapid symptom resolution. The mean hospital stay was 8.2 days. No recurrence or mortality was observed during follow-up.

Conclusion

This study highlights the importance of early diagnosis and individualized treatment in optimizing outcomes for ALA. The high rate of jaundice underscores the need for routine liver function evaluation. A structured approach, especially the use of percutaneous drainage in larger abscesses, was associated with excellent recovery. Community-based alcohol cessation initiatives may reduce ALA incidence in endemic regions.

## Introduction

An amebic liver abscess (ALA), the most severe extraintestinal manifestation of amebiasis caused by Entamoeba histolytica, remains a significant cause of morbidity in endemic regions, particularly in low- and middle-income countries where poor sanitation and high alcohol consumption are prevalent [1]. Globally, invasive amoebiasis affects an estimated 35-50 million individuals annually, resulting in approximately 55,000 deaths [1].

Despite advancements in diagnostics and therapeutics, delayed recognition and inappropriate management continue to pose major clinical challenges. Complications such as abscess rupture, peritonitis, and sepsis remain frequently encountered [2].

ALA develops following the hematogenous spread of E. histolytica trophozoites from the intestinal lumen to the liver, where they induce hepatocellular necrosis and abscess formation. The right hepatic lobe is most commonly affected, attributed to its dominant portal venous supply. The disease primarily affects adult males, largely due to alcohol-induced hepatic dysfunction and impaired immune responses, which facilitate E. histolytica invasion [3].

Alcohol is a well-established risk factor, as chronic consumption disrupts mucosal barriers, compromises gut integrity, and increases susceptibility to amebic translocation. Malnutrition and metabolic disorders further worsen the disease course by weakening host immunity [4].

The clinical presentation of ALA is often nonspecific. While fever, right upper quadrant pain, and hepatomegaly are common, atypical manifestations may lead to misdiagnosis and treatment delays. Notably, jaundice has emerged as a key marker of advanced disease due to hepatobiliary involvement, such as bile duct compression, hepatocellular injury, or secondary cholangitis. Though previously reported in 30-40% of cases, newer data suggest an even higher prevalence in endemic areas, warranting routine liver function evaluation [5].

Treatment strategies have shifted from antibiotic monotherapy to multimodal approaches incorporating percutaneous interventions for larger or refractory abscesses [5]. However, the optimal approach remains debated, particularly for medium-sized abscesses, where the timing and modality of intervention are not standardized. While needle aspiration and pigtail catheter drainage are both used, clinical decisions often depend on institutional preferences rather than unified guidelines. These management uncertainties persist despite recent advances in imaging, drainage techniques, and risk stratification.

This study aims to analyze the clinical profile, risk factors, hepatobiliary involvement, and treatment outcomes in ALA, using a structured, size- and severity-based approach. By identifying prognostic indicators and evaluating treatment efficacy, we aim to inform evidence-based decision-making and contribute region-specific data to the ongoing global effort to reduce ALA burden.

## Materials and methods

Study design and setting

This prospective observational study was conducted between 2022 and 2024 at the Department of Surgery, Dr. Pinnamaneni Siddhartha Institute of Medical Sciences and Research Foundation, a tertiary care center with a dedicated hepatobiliary unit. Ethical clearance was obtained from the Institutional Ethics Committee, and written informed consent was obtained from all participants.

Patient selection

Patients aged 18 years or older with clinical, imaging, and laboratory findings suggestive of ALA were enrolled. The diagnosis was based on characteristic symptoms, ultrasound findings, and supportive laboratory parameters. Patients with liver abscesses secondary to malignancy, immunocompromised status (HIV/AIDS, post-transplantation, and chronic steroid use), ascites, or ruptured abscesses extending into the peritoneal, pericardial, or pleural cavities were excluded.

Data collection and clinical assessment

Demographic details including age, sex, comorbidities, and relevant risk factors were recorded. Clinical features such as fever, right upper quadrant pain, jaundice, hepatomegaly, and gastrointestinal symptoms were documented. Detailed histories regarding alcohol consumption, nutritional status, and metabolic disorders were obtained.

Laboratory and imaging evaluation

All patients underwent a comprehensive laboratory evaluation including complete blood count, liver and renal function tests, coagulation profile, viral markers (HBV, HCV, HIV), serum albumin, and alkaline phosphatase (ALP) levels. The diagnosis was primarily based on ultrasonography, with contrast-enhanced computed tomography (CECT) performed selectively to assess abscess size and hepatobiliary involvement.

Management approach

All patients received empirical intravenous antibiotic therapy on admission. The standard regimen included metronidazole (750 mg three times daily for 10-14 days) and amoxicillin-clavulanate for 14 days. Broad-spectrum antibiotics (e.g., meropenem or third-generation cephalosporins with anaerobic coverage) were added if the secondary pyogenic infection was suspected. Antibiotic regimens were adjusted based on culture sensitivity results.

The percutaneous intervention was guided by abscess size and clinical response. Abscesses smaller than 5 cm were treated conservatively unless symptoms persisted. Intermediate-sized abscesses were managed with needle aspiration, while larger abscesses (>5 cm) were treated with ultrasound-guided pigtail catheter drainage. Aspirated material was sent for microscopy, culture, and sensitivity testing.

Patients with ruptured abscesses into the pleural or peritoneal cavity underwent additional interventions such as intercostal drainage or peritoneal tapping. Surgical intervention was considered only in refractory cases unresponsive to optimal medical and percutaneous treatment.

Follow-up and outcome assessment

All patients were followed for three months after discharge. Follow-ups were conducted every two weeks during the first two months and monthly thereafter. Treatment success was defined as the resolution of symptoms, a significant reduction in abscess size on ultrasound, and the absence of recurrence during the follow-up period.

Statistical analysis

This was a descriptive study; therefore, intergroup statistical comparisons (e.g., ANOVA and chi-square) were not performed. Although statistical methods were initially considered, the sample size and uniform outcomes (e.g., 100% recovery across all treatment modalities) limited the value of such comparisons. Although statistical comparisons were not performed in this study due to uniformity of outcomes and small sample size, a p-value <0.05 is generally considered significant in comparative clinical research.

## Results

This prospective study included 50 patients diagnosed with ALA. The mean age was 45.76 ± 10.2 years, with the highest prevalence observed in the 31-40 years age group (15 patients, 30%). A marked male predominance was noted, with 44 patients (88%) being male and six patients (12%) female, aligning with global trends that attribute higher ALA incidence among males to lifestyle and occupational risk factors. Alcohol consumption was present in 49 patients (98%), highlighting its central role as a predisposing factor. Chronic alcohol intake is known to disrupt intestinal mucosal barriers and impair immune function, promoting hepatic invasion by E. histolytica. Additionally, 24 patients (48%) were malnourished, and 21 patients (42%) were anemic, reinforcing the contribution of nutritional deficiencies in ALA pathogenesis (Table 1).

**Table 1:** Baseline demographic and risk factor profile of patients with amebic liver abscess Data are presented as mean ± SD for continuous variables and numbers (%) for categorical variables. No statistical comparisons were performed; in comparative studies, a p-value <0.05 is typically considered significant.

Characteristic	Value (n = 50)
Mean Age (years)	45.76 ± 10.2
Most Affected Age	31-40 years (n = 15, 30%)
Male Patients	44 (88%)
Female Patients	6 (12%)
Alcohol Consumption	49 (98%)
Malnutrition	24 (48%)
Anemia	21 (42%)

Most patients (39, 78%) presented within two weeks of symptom onset, while 11 (22%) had a delayed presentation. Delayed presentation has been associated with larger abscess size, increased complications, and longer hospital stays, emphasizing the need for early detection. The most common symptom was right upper quadrant pain (42 patients, 84%), followed by fever (39 patients, 78%), jaundice (25 patients, 49%), and diarrhea (16 patients, 32%) (Table 2).

**Table 2:** Baseline demographic and risk factor profile of patients with amebic liver abscess Data are expressed as n (%). No statistical comparisons were performed, and test values were not applicable; in comparative studies, a p-value <0.05 is typically considered significant.

Clinical Feature	Frequency (n = 50)
Right Upper Quadrant Pain	42 (84%)
Fever	39 (78%)
Jaundice	25 (49%)
Diarrhea	16 (32%)

Hypoalbuminemia (<3.5 g/dL) was present in 35 patients (70%), indicating systemic inflammation and hepatic dysfunction. Elevated ALP (ALP >200 IU/L) was observed in 21 patients (42%), with 18 (36%) having ALP levels between 200 IU/L and 400 IU/L (Table 3). These findings reflect cholestasis or biliary obstruction associated with a larger abscess burden.

**Table 3:** Laboratory parameters in patients with amoebic liver abscess Data are expressed as n (%). No statistical comparisons were performed, and test values were not applicable; in comparative studies, a p-value <0.05 is typically considered significant.

Parameter	Normal (n, %)	Abnormal (n, %)
Serum Albumin (<3.5 g/dL)	15 (30%)	35 (70%)
ALP (>200 IU/L)	29 (58%)	21 (42%)

Ultrasound confirmed right lobe involvement in 47 patients (94%), consistent with known vascular anatomy. Hepatomegaly was present in 20 patients (40%). The mean abscess diameter was 6.8 cm (range: 3-12 cm). Solitary abscesses were identified in 36 patients (72%) and multiple abscesses in 14 (28%) (Table 4). Larger or multiloculated abscesses were often associated with delayed presentation or failure of conservative management.

**Table 4:** Radiological findings observed in amebic liver abscess cases Data are expressed as n (%). No statistical comparisons were performed, and test values were not applicable; in comparative studies, a p-value <0.05 is typically considered significant.

Imaging Finding	Prevalence (n, %)
Right Lobe Involvement	47 (94%)
Left Lobe Involvement	3 (6%)
Hepatomegaly	20 (40%)
Single Abscess	36 (72%)
Multiple Abscesses	14 (28%)

Management was guided by abscess size and clinical severity. Antibiotic therapy alone led to full resolution in 20 patients (40%), predominantly those with abscesses <5 cm in diameter and without complications. Needle aspiration was performed in 13 patients (26%) and pigtail catheter drainage in 17 (34%) (Table 5). Pigtail drainage was preferred in patients with large or non-responding abscesses, resulting in rapid symptom relief. The average hospital stay was 8.2 days, longer in those undergoing interventional procedures. No mortality or recurrence was reported within three months of follow-up.

**Table 5:** Treatment modalities and clinical outcomes in amebic liver abscess patients Data are presented as numbers (%). No statistical comparisons were performed due to uniform outcomes; in comparative studies, a p-value <0.05 is typically considered significant.

Treatment Modality	Prevalence (n, %)	Clinical Response
Antibiotics Alone	20 (40%)	100% recovery
Needle Aspiration	13 (26%)	100% recovery
Pigtail Catheter Drainage	17 (34%)	100% recovery

All patients were monitored for three months post-discharge, with no cases of recurrence or mortality observed. Serial ultrasound follow-up confirmed complete resolution of the abscess in all patients, highlighting the effectiveness of structured management approaches.

These findings emphasize that early diagnosis and tailored intervention are key to optimizing patient outcomes. Patients with smaller abscesses (<5 cm) benefited from medical therapy alone, whereas larger abscesses required percutaneous drainage for faster recovery and reduced risk of complications.

## Discussion

ALA remains one of the most significant infectious hepatic diseases globally, with over 50 million cases and approximately 55,000 deaths annually [1]. Our study reinforces the urgent need for structured management strategies in endemic settings, particularly given the overwhelming influence of modifiable risk factors such as chronic alcohol consumption and malnutrition. In our cohort, 98% of patients reported alcohol use, highlighting its direct role in disease pathogenesis. Alcohol induces gut mucosal injury, increases intestinal permeability, and impairs hepatic immunity, thereby facilitating the hematogenous spread of E. histolytica to the liver. This strong epidemiological and pathophysiological link justifies the integration of alcohol cessation interventions into preventive liver health programs [6].

A notable clinical observation in our study was the high incidence of jaundice (49%), exceeding the 30-40% typically reported in prior literature. This finding underscores the evolving nature of ALA in endemic populations and may indicate more aggressive hepatobiliary involvement in alcohol-abusing or late-presenting patients. Mechanistically, jaundice in ALA results from bile duct compression by the expanding abscess cavity, direct hepatocellular necrosis, or secondary cholangitis caused by inflammatory infiltrates. Elevated serum bilirubin and ALP in our patients further support this explanation. These results advocate for routine liver function testing at presentation, not only for diagnostic clarity but also for early risk stratification and triage to interventional care [7].

Our structured, size-based treatment algorithm was central to the excellent outcomes observed. Patients with smaller abscesses (<5 cm) responded well to antibiotic therapy alone, while those with abscesses >5 cm achieved faster resolution through percutaneous drainage. Among drainage modalities, pigtail catheterization appeared superior to needle aspiration in terms of symptom relief and abscess resolution. The sustained decompression provided by catheter drainage prevents re-accumulation of necrotic material, minimizes abscess expansion, and accelerates clinical improvement, outcomes that are pathophysiologically rational and supported by prior interventional literature [8]. These findings suggest that for borderline abscesses (e.g., 4.5-6 cm), early catheter drainage may offer a clinical advantage, even when guidelines favor conservative management.

In addition to hepatobiliary involvement, our study highlighted significant systemic effects. Hypoalbuminemia was observed in 70% of patients and anemia in 42%. These abnormalities likely result from a combination of chronic inflammation, hepatic synthetic dysfunction, and protein-losing enteropathy. Furthermore, 48% of patients were malnourished, a state that compromises immune function and may predispose to more aggressive disease or delayed recovery. These observations underscore the importance of incorporating nutritional assessment and support as integral components of ALA management, especially in resource-limited settings.

From a public health standpoint, our findings reiterate the need for early screening and targeted intervention. The combined burden of alcohol use, malnutrition, and late presentation creates a clinical profile that demands a proactive approach. Community-based programs focusing on alcohol awareness, nutritional education, and early ultrasonographic screening may help shift the disease profile toward earlier, less severe presentations and reduce the need for invasive interventions.

This study, while promising, is not without limitations. The sample size was modest, and due to the uniform clinical outcomes, inferential statistical tests were not performed. The single-center design may also limit generalizability. Additionally, the lack of molecular diagnostics, such as PCR, may have resulted in the inclusion of misclassified pyogenic liver abscess cases. Future multi-center studies with larger cohorts and access to advanced diagnostics are needed to validate our findings. Moreover, prospective evaluations of alcohol cessation programs and nutritional interventions in high-risk populations could significantly alter the incidence and outcomes of ALA [9-11].

## Conclusions

This prospective study highlights the evolving clinical profile of ALA, particularly the high prevalence of hepatobiliary involvement such as jaundice, which was observed in 49% of our cohort. These findings suggest that liver function abnormalities may serve as early indicators of disease severity and should be routinely evaluated for effective risk stratification. The structured, size-based management approach employed, antibiotics for abscesses <5 cm and percutaneous drainage for larger or non-resolving lesions, resulted in excellent clinical outcomes, with no recurrence or mortality during follow-up. Notably, pigtail catheter drainage was associated with faster symptom resolution, reinforcing its clinical utility.

The near-universal association of ALA with alcohol consumption (98%) and the high burden of malnutrition emphasize the importance of addressing modifiable risk factors. Integrating alcohol cessation and nutritional support into liver disease prevention strategies may significantly reduce disease incidence in endemic regions. Although limited by a modest sample size and the absence of inferential statistical analysis, this study provides practical insights into optimizing ALA management in resource-constrained settings. Future multicenter studies incorporating larger cohorts and molecular diagnostics are needed to validate these findings and inform the development of standardized, evidence-based treatment protocols.
